# Repetitive motor cortex stimulation reinforces the pain modulation circuits of peripheral neuropathic pain

**DOI:** 10.1038/s41598-017-08208-2

**Published:** 2017-08-11

**Authors:** Myeounghoon Cha, Sun Woo Um, Minjee Kwon, Taick Sang Nam, Bae Hwan Lee

**Affiliations:** 1 Department of Physiology, Yonsei University College of Medicine, Seoul, 03722 Republic of Korea; 2 Brain Korea 21 PLUS Project for Medical Science, Yonsei University College of Medicine, Seoul, 03722 Republic of Korea

## Abstract

Recent evidence indicates that motor cortex stimulation (MCS) is a potentially effective treatment for chronic neuropathic pain. However, the neural mechanisms underlying the attenuated hyperalgesia after MCS are not completely understood. In this study, we investigated the neural mechanism of the effects of MCS using an animal model of neuropathic pain. After 10 daily sessions of MCS, repetitive MCS reduced mechanical allodynia and contributed to neuronal changes in the anterior cingulate cortex (ACC). Interestingly, inhibition of protein kinase M zeta (PKMζ), a regulator of synaptic plasticity, in the ACC blocked the effects of repetitive MCS. Histological and molecular studies showed a significantly increased level of glial fibrillary acidic protein (GFAP) expression in the ACC after peripheral neuropathy, and neither MCS treatment nor ZIP administration affected this increase. These results suggest that repetitive MCS can attenuate the mechanical allodynia in neuropathic pain, and that the activation of PKMζ in the ACC may play a role in the modulation of neuropathic pain via MCS.

## Introduction

When the sensory nervous system is affected by injury or disease, it often leads to a sense of numbness or lack of sensation. Neuropathic pain is thought to be associated with these types of abnormal peripheral and central nerve problems, which can lead to the development of a chronic neuropathic pain state. Recent studies have suggested that the development of neuropathic pain involves not only neurons but also glial cells, including astrocytes and microglia, which interact with neurons and thereby modulate pain transmission under pathophysiological conditions^1–3^. Increased peripheral sensory nerve activity induces multiple trans-synaptic modifications that extend to the central nervous system (CNS). Furthermore, persistent chronic pain induces significant functional and structural changes in the nervous system^4^. These new synaptic formations in the CNS underlie the plasticity of neurons. Neuropathic pain-induced synaptic plasticity has been documented in many cortical regions associated with pain perception^5, 6^. A recent report demonstrated that an elevation in astrocytic activity initiates increased synaptic remodeling in the brain^7^. The strengthening of the synaptic interactions between specific cells in the CNS affected the formation of a new memory. When LTP is generated, the number of surrounding astrocytes increases to supply sufficient energy for the newly generated synapses^6^. Therefore, the degree of astrocytic hypertrophy, which of can be determined by measuring the number of and assessing the shape of astrocytes, can be used as direct or indirect evidence of activated synaptic plasticity^2, 8^.

Since its initial publication in the early 1990s, epidural motor cortex stimulation (MCS) using surgically implanted electrodes has been shown to be capable of producing long-term analgesia in approximately half of the patients with chronic neuropathic pain resistant to medication^9^. MCS is easier to implement for pain modulation than other surgical methods, such as direct nerve stimulation and neurectomy, and it can be considered an alternative treatment for pain control^10^. Recent studies have reported that pain relief occurs progressively after the onset of MCS and persists after the stimulation has stopped^11–13^. This effect of MCS can last from minutes to days in some patients and suggests that MCS could potentially serve as a therapy for the treatment of resistant neuropathic pain^14, 15^. Furthermore, repetitive stimulation of the motor cortex induces homeostatic plasticity as a means of stabilizing the properties of neuronal circuits in the brain^16, 17^. However, the underlying mechanism of MCS in pain modulation is poorly understood.

Recent studies have described the anterior cingulate cortex (ACC) as a cortical area in the brain involved with pain, possibly including both perception and modulation via neural plasticity^18, 19^. However, despite studies demonstrating that ACC projection is deeply related to the motor cortex, the underlying mechanism of MCS in pain modulation is poorly understood^18^. The activation of astrocytes and/or astrogliosis is one of the changes that has been observed in the ACC during chronic pain induced by nerve injury^20, 21^. In addition, nerve injury manifests as an increased expression of astrocytic markers, such as glial fibrillary acidic protein (GFAP), in the ACC^8^. Evidence from previous reports shows that astrocytes perform various functions, including the biochemical support of endothelial cells that form the blood–brain barrier, provision of nutrients to nervous tissue, maintenance of extracellular ion balance, and a role in the repair and scarring process of the brain and spinal cord following traumatic injuries^22–24^. Astrocytes are the most numerous non-neuronal cells in the brain involved in the modulation of neuronal activities, such as extracellular and synaptic cleft neurotransmitter level regulation and the release of neuroactive molecules^18^. Moreover, recent studies have suggested that astrocytes play an important role in the synaptic cleft and in the interactions between pain-transmitting neurons and other neurons^5, 7^. This is because astrocytes can detect neuronal activity and release chemical transmitters, which in turn control synaptic activity^19, 22^.

Thus, we hypothesized that repetitive MCS may induce analgesic effects on chronic neuropathic pain by inducing a modification in synaptic connections, resulting in the attentuation of mechanical hypersensitivity in neuropathic pain. The aim of this study was to examine the altered synaptic connection of the ACC in a rat model of neuropathic pain and MCS-induced behavioral modifications. Our results implicate the potential role of cortical astrocytes and ACC structural synaptic plasticity in mechanical hyperalgesia and contribute to the understanding of the mechanism of MCS-induced analgesic effects in an animal model of neuropathic pain.

## Results

### Repetitive MCS attenuated pain behavior associated with neuropathic pain

**Figure 1:**
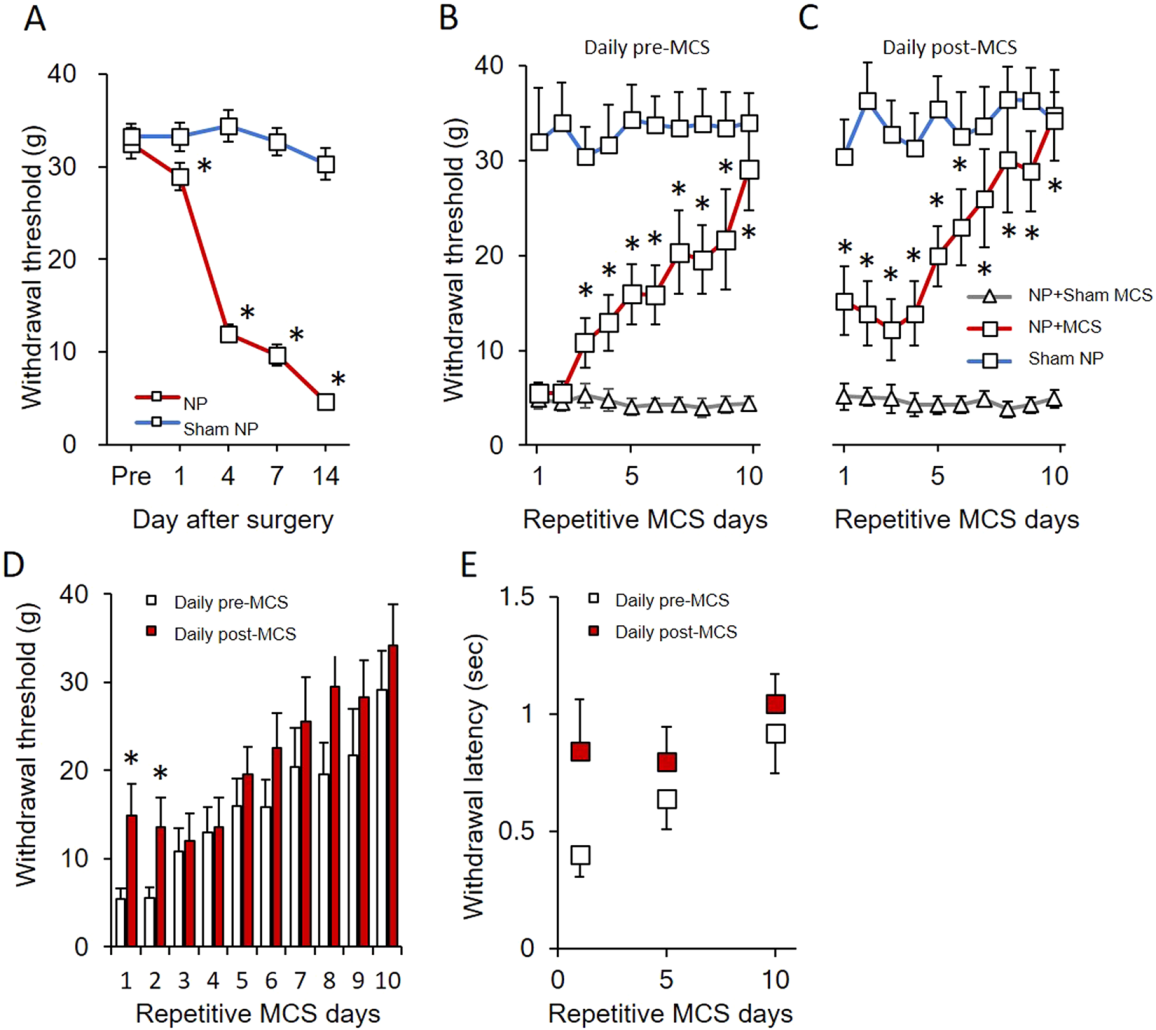
MCS effects on neuropathic pain. (**A**) Development of mechanical allodynia after nerve injury. On days 1, 4, 7, and 14 after surgery, neuropathic rats showed a significantly decreased mechanical withdrawal threshold compared with the sham-operated group (**B**) Comparison of the daily pre-MCS behavioral tests. The mechanical threshold changes between groups were compared with the daily pre-MCS session. From the second MCS, the withdrawal threshold gradually increased until the last day of MCS. (**C**) Comparison of the daily post-MCS behavioral tests. The mechanical threshold changes between groups were compared with the daily post-MCS session. The withdrawal thresholds were immediately elevated after MCS and gently increased. (**D**) Comparison of the withdrawal threshold changes at daily pre/post-MCS in neuropathic pain. After MCS, the mechanical threshold became insensitive and the thresholds increased with daily MCS. When comparing the daily pre/post-MCS, the thresholds of the post-MCS were higher than the pre-MCS. (**E**) Comparison of the withdrawal latency changes according to repetitive MCS. The latency was immediately elevated after MCS and gradually increased after repetitive MCS (Daily pre-MCS: ρ value = 0.753, *P* = 0.03; Daily post-MCS: ρ value = 0.28, *P* = 0.13). *P < 0.05 compared with NP + sham MCS.

We have previously shown that tibial and sural nerve ligation in rats produced mechanical and thermal hypersensitivity^25^. The withdrawal threshold and latency induced by electrical von Frey stimulation were measured in peripheral nerve-injured rats. As shown in Fig. 1A, the withdrawal thresholds in neuropathic pain (NP) rats significantly decreased after nerve injury. The measured values of the withdrawal response in NP rats were significantly reduced from the day following nerve injury (*P* < 0.05 vs. sham operated rats). To observe the effect of repetitive MCS on neuropathic pain, 10 repetitive daily sessions of MCS were performed on NP and sham NP rats 14 days after the operation. Figure 1B and C shows the daily withdrawal threshold changes pre- and post-MCS in different groups (NP + sham MCS, NP + MCS, and sham NP). Although MCS in NP animals showed immediately reduced hypersensitivity on the first day post-MCS, the reduced hypersensitivity did not exist the day after the first MCS. However, the withdrawal threshold of MCS on NP rats gradually increased following repetitive stimulation of the motor cortex (*P* < 0.05 vs. NP + sham MCS). To evaluate the effects of MCS, 10 daily sessions of sham MCS (without electric stimulation) were performed in neuropathic rats. However, sham MCS on neuropathic animals did not affect the withdrawal threshold. Figure 1D shows the comparison of withdrawal threshold changes between daily pre- and post-NP + MCS on neuropathic rats. On the first and second days of MCS, the withdrawal thresholds of pre- and post-MCS were significantly different. The mechanical withdrawal thresholds of MCS-treated rats continuously improved until the final day of MCS. To determine the effects of repetitive MCS on the withdrawal latency response, we measured the withdrawal latency at days 1, 5, and 10 of pre- and post-NP + MCS (Fig. 1E). Repetitive MCS attenuated the behavioral sensitivity in neuropathic rats. The withdrawal latency of the pre-NP + MCS notably increased during repetitive MCS (σ value = 0.753, *P* = 0.03). However, the withdrawal latency of the post-NP + MCS did not significantly change (σ value = 0.28, *P* = 0.13).

### Effects of ZIP on MCS-induced analgesia

**Figure 2:**
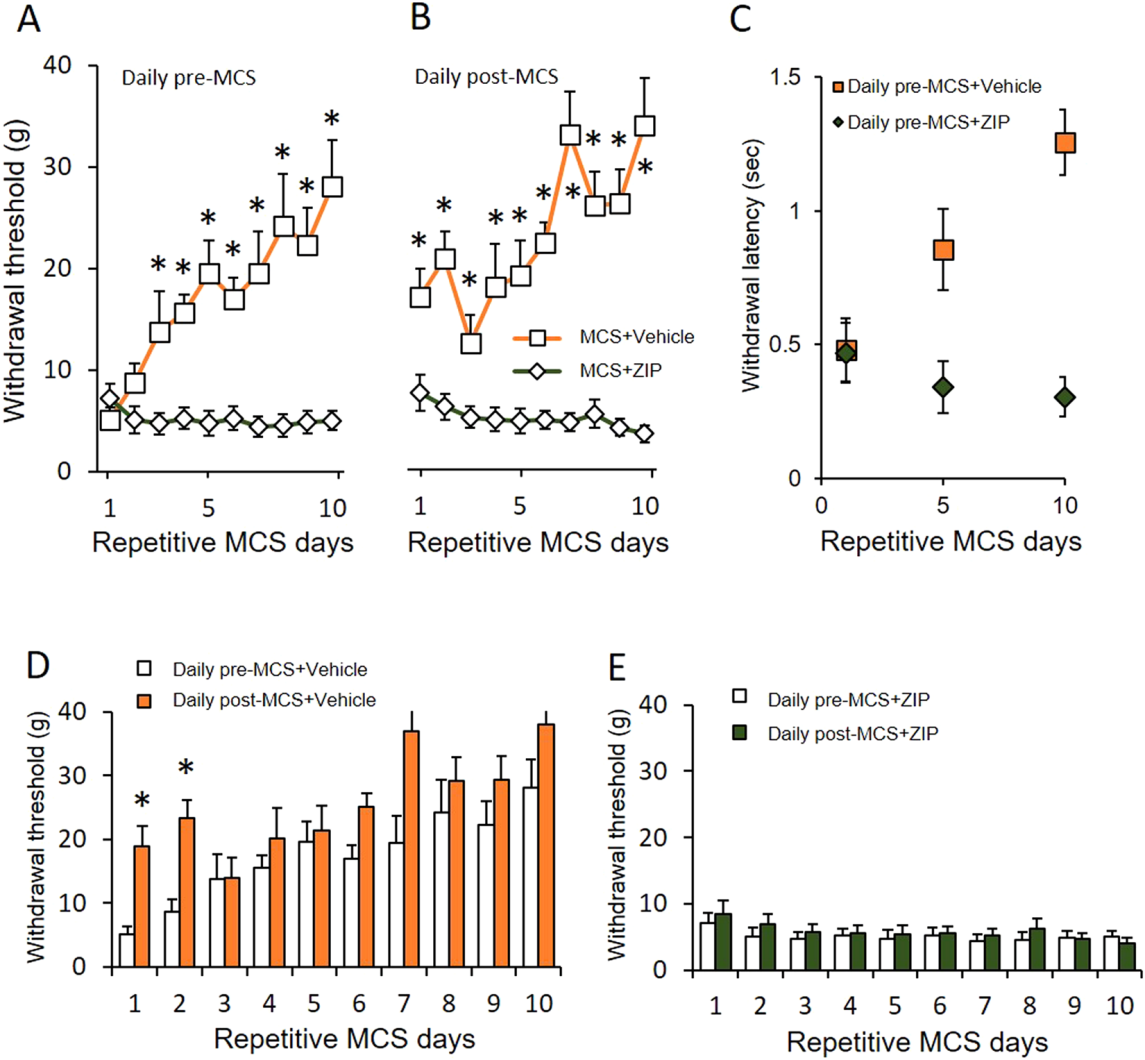
ZIP effects on the MCS-treated neuropathic pain. (**A**) Comparison of ZIP and vehicle injections with pre-MCS in neuropathic rats. Mechanical allodynia developed in the MCS with vehicle injection group. However, ZIP injection group did not show significant changes. (**B**) Comparison of ZIP and vehicle injections with post-MCS in neuropathic rats. Vehicle injection did not influence the effects of MCS. However, the effects of MCS were completely blocked by ZIP injections in the ACC. (**C**) Comparison of the withdrawal latency changes between MCS + Vehicle and MCS + ZIP injection rats. Latency of vehicle-injected rats was gradually elevated after repetitive MCS (Daily pre-MCS in MCS + Vehicle rats: ρ value = 0.8143, *P* = 0.04). (**D**) Comparison of pre- and post-withdrawal threshold changes in the MCS with the vehicle injection group. Vehicle injection did not affect the MCS effects. (**E**) Comparison of pre- and post-withdrawal threshold changes in the MCS with the ZIP injection group. ZIP injection abolishes the effects of MCS.

To determine whether there was MCS-induced neural plasticity in the ACC, we performed microinjection of ζ-pseudosubstrate inhibitory peptide (ZIP, a selective inhibitor of PKMζ) in the ACC region. To quantify the effects of the ZIP on MCS treatment in neuropathic rats, ZIP and vehicle injections were compared. To avoid MCS-induced neural plasticity, repetitive daily sessions of MCS were followed by concomitant ZIP injections. As shown in Fig. 2A and B, MCS-induced analgesic effects were not observed in MCS + ZIP injections in NP rats. Additionally, withdrawal latency and mechanical thresholds did not change with the ZIP injections in the NP + MCS group (Fig. 2C). However, vehicle injections in NP with MCS rats showed significantly increased withdrawal thresholds immediately after MCS, which continuously improved until the final day of repetitive MCS. Figure 2D indicates the withdrawal threshold changes between daily pre- and daily post-MCS in the vehicle injection rats, whereas Fig. 2E presents the withdrawal threshold changes between pre- and post-MCS stimulation in the ZIP injection group. The results of the ZIP and vehicle injections along with MCS in neuropathic rats indicate that the ZIP injection could prevent MCS-induced analgesic effects in neuropathic rats.

**Figure 3:**
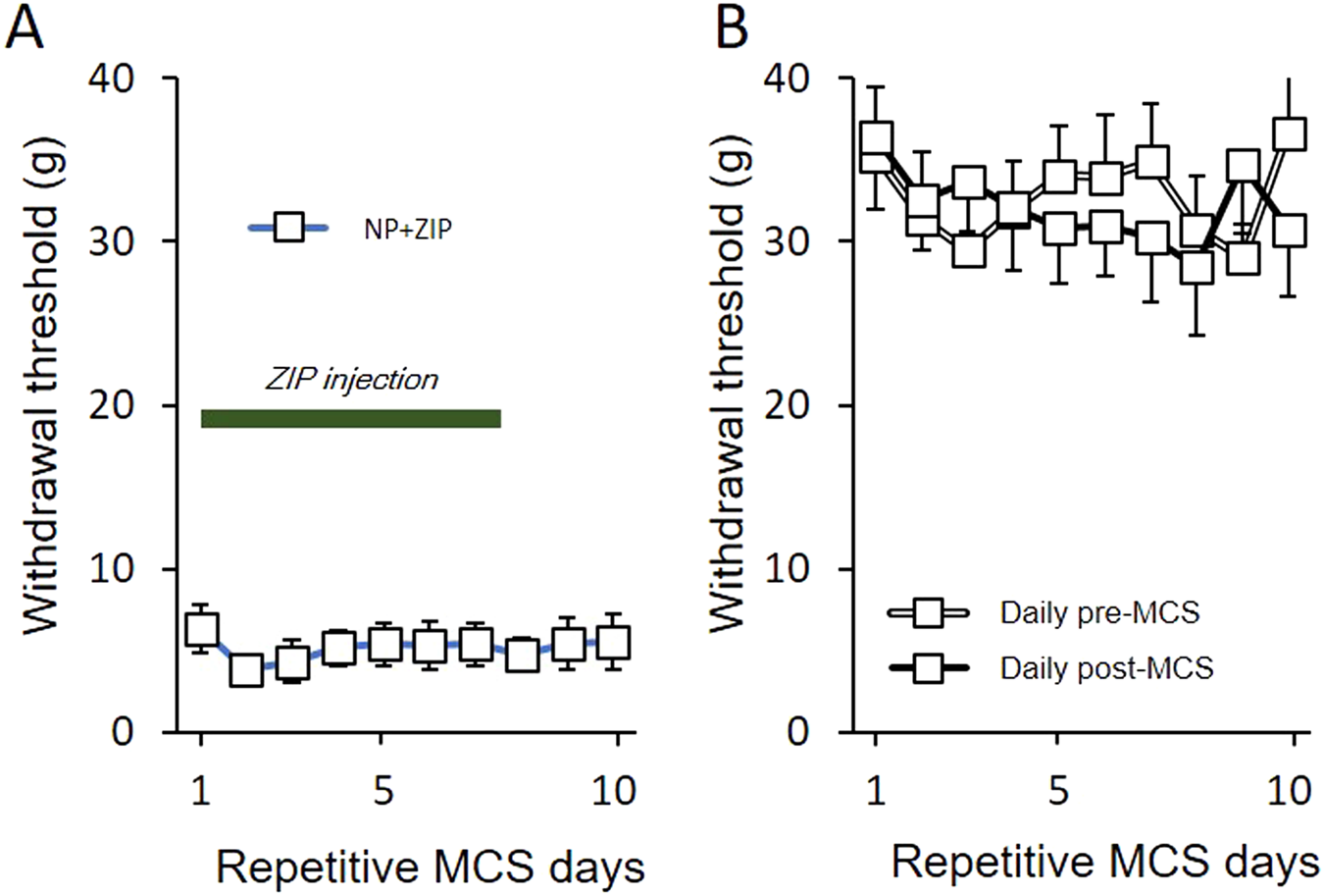
The effects of ZIP injection on neuropathic rats and MCS on naïve rats. (**A**) The effects of ZIP injection on neuropathic rats. In the behavioral test after 14 days in the NP rats, the withdrawal thresholds did not significantly change between the duration of ZIP injection and the end of ZIP injection days. (**B**) For evaluation of the effects of MCS on the control (non-injured) rats, the mechanical thresholds were measured daily pre- and post-MCS. In the comparison of pre- and post-MCS, the withdrawal thresholds did not change over the duration of MCS.

To investigate the ZIP effects on nerve injury-induced mature neural plasticity, a ZIP solution was injected 14 days after nerve injury in rats once they fully developed neuropathic pain (without MCS). Figure 3A shows that the ZIP injection after neuropathic surgery in rats did not lead to withdrawal changes during the duration of the ZIP injection. This result could indicate that ZIP does not affect the mature state of neural plasticity. In addition, MCS was performed on control (naïve) rats to examine MCS-induced paresthesia (Fig. 3B). Comparison of daily pre- and daily post-MCS on the control rats did not show significant threshold changes during the MCS session, indicating that MCS did not interfere with the mechanical threshold in naïve rats.

**Figure 4:**
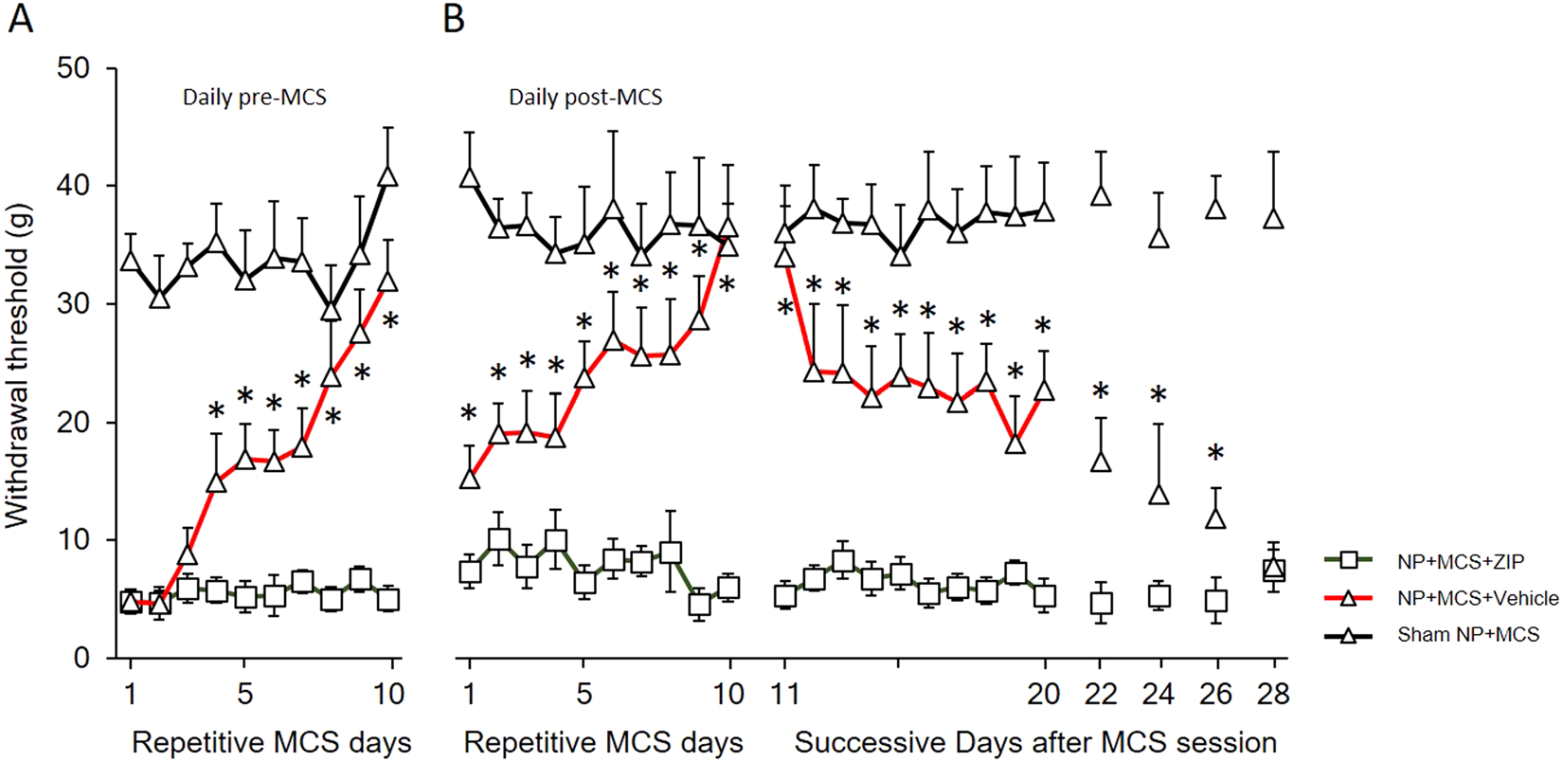
Long-term effects of MCS. (**A**) Comparison of the effects of daily pre-MCS in different conditions. Neuropathic pain rats with MCS show gradually increased withdrawal thresholds up to the 10^th^ day after MCS. (**B**) To evaluate the duration of the effects of daily post-MCS, the mechanical thresholds were measured daily until the 28^th^ day after MCS. The effects of MCS gradually decreased until the 17^th^ day after termination of MCS.

To observe the long-term effects of MCS, we conducted a daily behavioral test until the effect of repetitive MCS disappeared. Figure 4A and B show the withdrawal threshold changes of MCS with vehicle injection in NP rats compared with MCS with ZIP injection in NP rats and sham NP with MCS rats. The withdrawal thresholds of daily post-MCS were significantly increased on the first day of MCS and continuously increased until the last day of MCS (*P* < 0.05 vs. NP + MCS + ZIP). After the end of the repetitive daily MCS session, mechanical threshold changes were observed until the MCS effects disappeared. The elevated withdrawal thresholds were gradually reduced and continued to decrease until 2 weeks after the end of the daily MCS sessions. This result implies that the effects of repetitive MCS last a long time after the end of stimulation.

**Figure 5:**
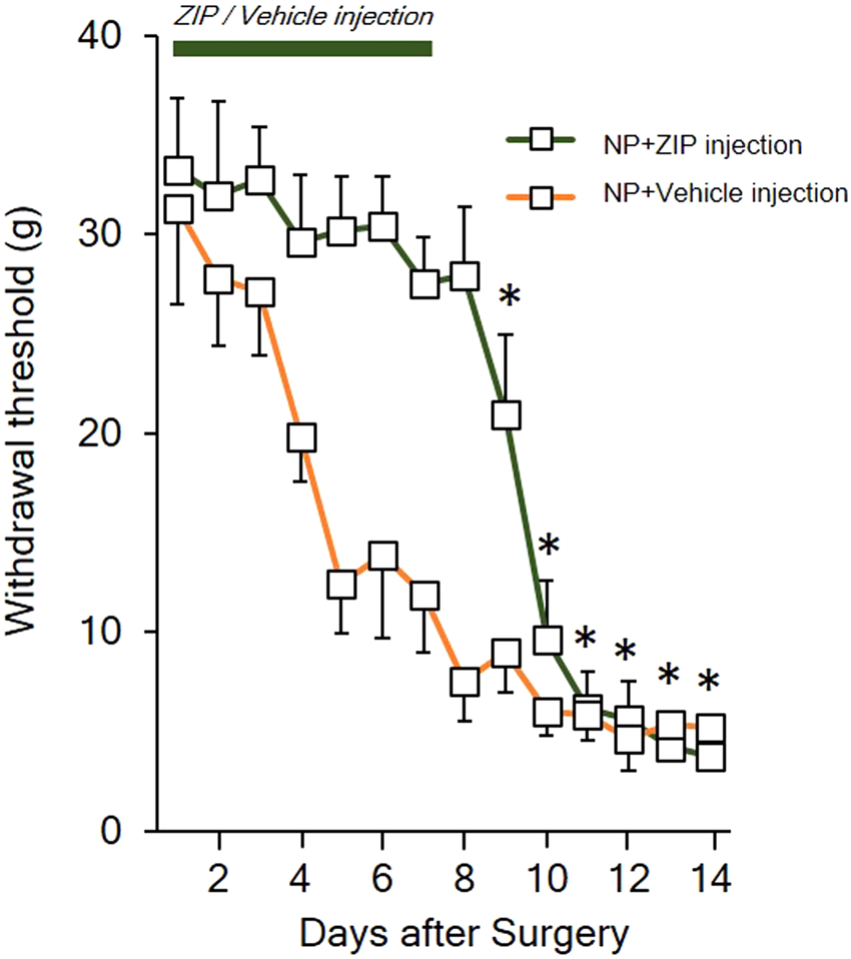
The effect of ZIP injections on the initial stage of neuropathic pain. To evaluate the effects of ZIP on the initial state of neuropathic pain, ZIP was injected into the ACC after neuropathic surgery. ZIP-injected rats showed a normal mechanical threshold up to 7 days after surgery. However, after the completion of the ZIP injection, the mechanical threshold decreased rapidly, which resulted in the development of neuropathic pain.

Additionally, we investigated whether the ZIP injection could inhibit the initial state of neuropathic pain development. After neuropathic surgery, cannulation surgery was performed on the same day as the microinjection of ZIP. The withdrawal thresholds of the NP rats injected with either ZIP or vehicle were compared. As shown in Fig. 5, the mechanical threshold changes were not significantly reduced during the ZIP injection period. However, the withdrawal threshold significantly decreased on the ninth day after surgery. The withdrawal threshold rapidly decreased until the 10^th^ day after surgery and continued to decrease. On the 12^th^ day after surgery, there was no significant difference in the withdrawal threshold in NP rats injected with ZIP or vehicle. These data show that the microinjection of ZIP into the ACC prevented the inhibition of the nociceptive threshold during its application (7 days) and delayed the onset of allodynia, which became similar to the vehicle group only on the 11th day.

### Changes in the level of GFAP immunoreactivity in the ACC of rats with neuropathic pain

**Figure 6:**
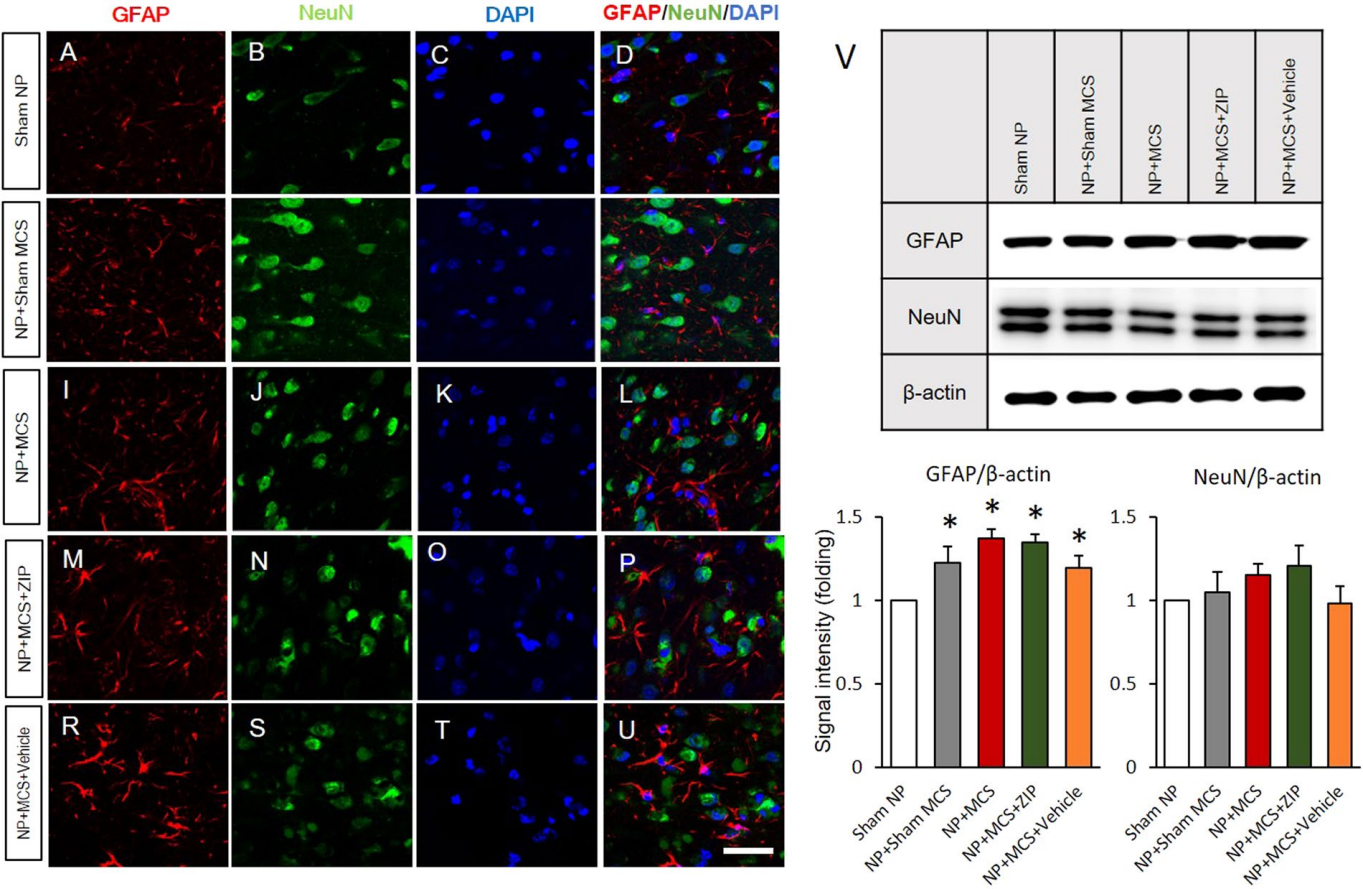
Comparison of neuronal and glial cell alterations in the ACC. Results of double staining of rat brain slices for the astrocyte marker GFAP and neuron marker NeuN are shown. DAPI counterstaining is indicated by blue fluorescence. (**A**–**D**) The expression of GFAP and NeuN in neuronal cells in sham NP rats is shown. (**E**–**U**) GFAP immunoreactivity in the ACC was elevated in all experimental groups. However, the expression levels of NeuN did not significantly change in all animal models used (scale bar, 50 μm). (**V**) Western blot analysis was used to detect GFAP and NeuN protein levels in the ACC. GFAP expression in the ACC significantly increased in the NP + Sham MCS, NP + MCS, NP + MCS + ZIP, and NP + MCS + Vehicle rats. Elevated NeuN expression was detected in NP + MCS and NP + MCS + ZIP rats, but significant changes were not observed. β-Actin was used as a loading control. *P < 0.05 compared with sham NP and n = 4 rats for each group.

The ACC is a key cortical area involved in chronic pain. We therefore used immunohistochemistry to observe changes in protein expression in neurons and glial cells in the ACC region. The analgesic behavioral changes were affected by long-term potentiation (LTP)-like synaptic modifications. Figure 6 shows the comparison of neuronal cell-specific protein expression in experimental groups. The expression of GFAP and NeuN in glial cells and neurons, respectively, in sham NP rats was used as a standard for comparison (Fig. 6A to D). We found that the population of astrocytes intensely increased on the 14^th^ day after nerve injury in NP rats (Fig. 6E). We performed NeuN and DAPI staining in the ACC region to observe the changes in the neuronal population caused by neuropathic pain (Fig. 6F and G). However, the expression of NeuN did not significantly change. In the merged figure, the astrocytes near the neuronal cells display increases in NeuN expression, dendritic length, cell body area and the number of nodes (Fig. 6H). Furthermore, we compared the neuronal cell-specific protein expression between NP + Sham MCS rats and NP + MCS rats. The dendritic length, cell body area, and number of nodes of astrocytes were not significantly elevated in NP + MCS rats (Fig. 6I), and NeuN expression and DAPI staining intensity also did not significantly increase. These data suggest that the effects of MCS on astrocyte-specific protein expression in the ACC region are difficult to define after mature synaptic changes at the end of experiments. When comparing MCS with ZIP injection and MCS with vehicle injection in the NP rats, the expression of astrocyte-specific proteins did not significantly change (Fig. 6M to U). These data suggest that astrocytes in the ACC are initially affected by nerve injury; however, it is difficult determine whether morphological changes are only induced by MCS. To measure changes in astrocytes, we evaluated GFAP and NeuN expression by western blotting (Fig. 6V). The levels of GFAP in the ACC significantly increased after nerve injury (Sham NP, 1; NP + Sham MCS, 1.27 ± 0.01; NP + MCS, 1.37 ± 0.05; NP + MCS + ZIP, 1.35 ± 0.04; NP + MCS + Vehicle, 1.20 ± 0.07). We also detected possible changes in NeuN expression. However, the regional and cellular distributions of NeuN did not differ between the sham NP and other groups (Sham NP, 1; NP + Sham MCS, 1.05 ± 0.11; NP + MCS, 1.14 ± 0.07; NP + MCS + ZIP, 1.20 ± 0.12; NP + MCS + Vehicle, 0.98 ± 0.11).

### Quantification of astrocytic hypertrophy

**Figure 7:**
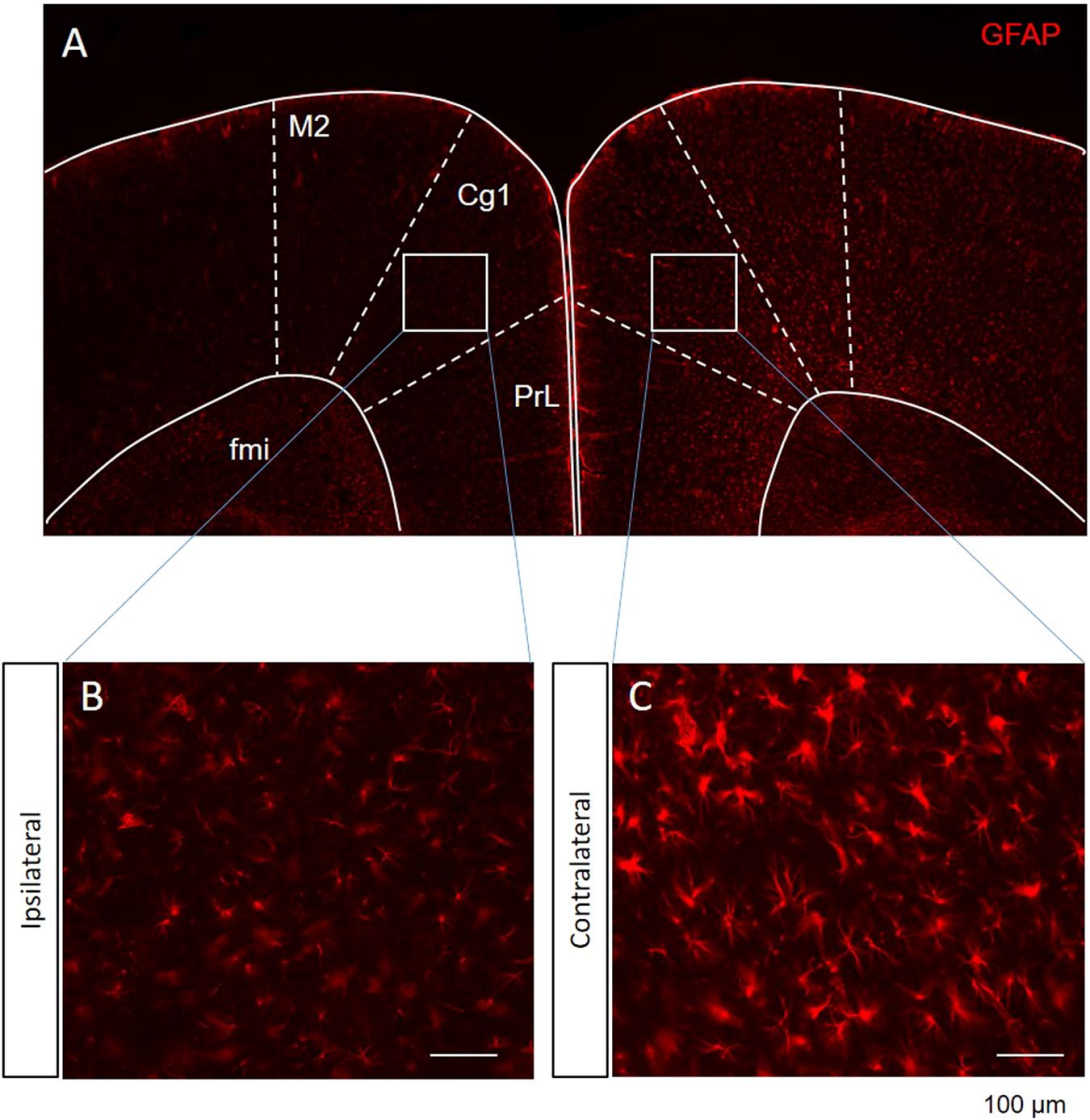
The morphological appearance of astrocytes in the ACC of NP rats. (**A**) Comparison of the different GFAP expression levels in the ipsilateral and contralateral sides of the ACC region. (**B**) Ipsilateral and (**C**) contralateral sides of the ACC in NP rats. (Scale bar, 100 μm; M2: secondary motor cortex, Cg1: cingulate cortex area 1, PrL: prelimbic cortex, fmi: forceps minor corpus callosum).

The morphological appearance of astrocytes in the ACC of NP rats showed strong hypertrophy expression (Fig. 7), whereas astrocytes from the ipsilateral side of the ACC in NP rats exhibited moderate hypertrophy compared with the contralateral side (Fig. 7B). Based on a general visual examination of the staining, the astrocyte-specific protein expression seems to be higher in the contralateral side compared with the ipsilateral side of nerve-injured rats (Fig. 7C). As the sham injury model is considered to be mild to moderate, we did not observe robust hypertrophy 14 days after nerve injury. Further characterization of the astrogliosis in the NP rats revealed that, although some of the hypertrophic astrocytes were in the deep layer of the ACC region, hypertrophic astrocytes were more often observed at the outer line of the forceps minor corpus callosum.

**Figure 8:**
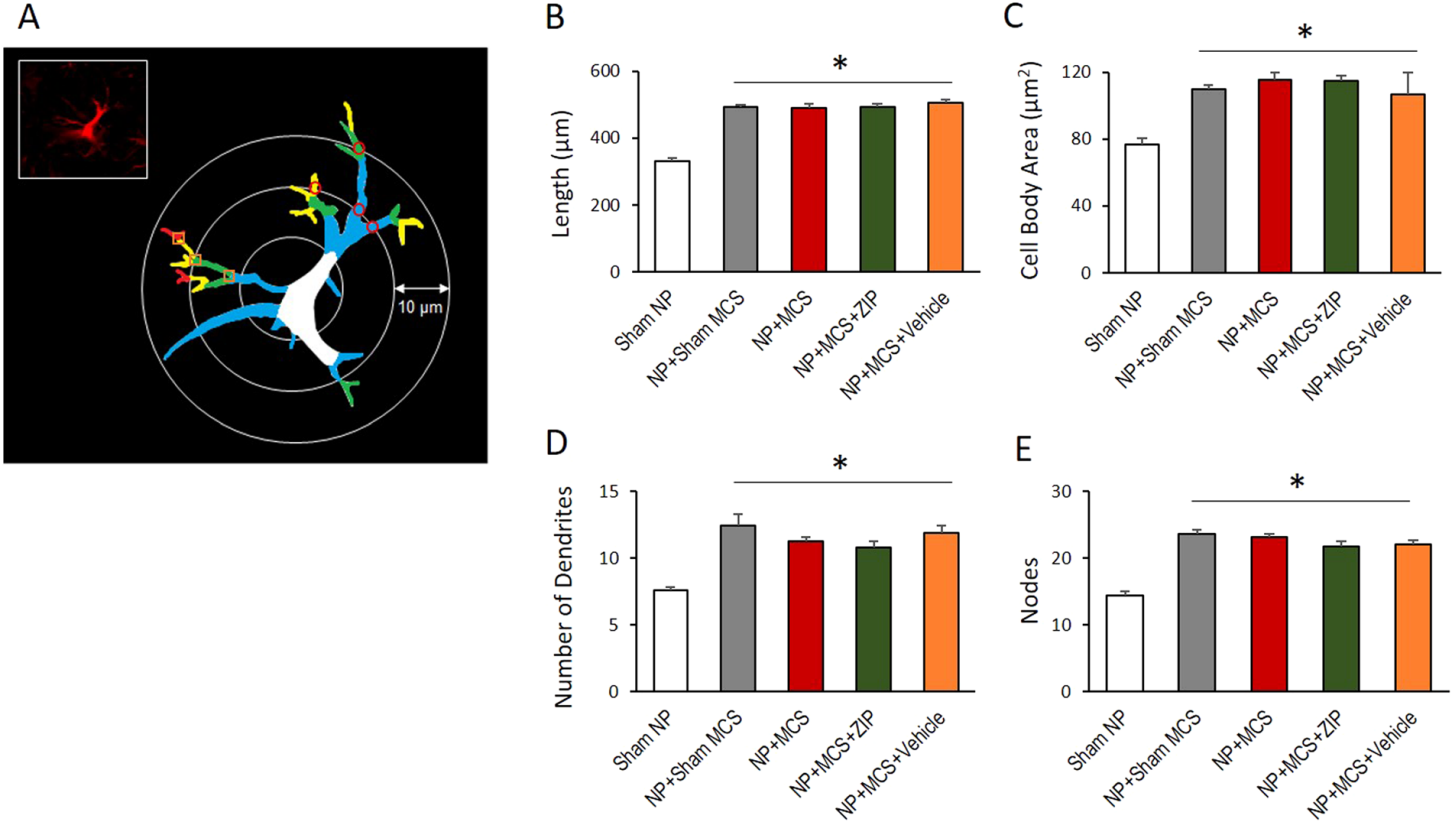
Neuropathic pain and MCS induce astrocytic hypertrophy. (**A**) Schematic of astrocytes and the parameters measured. Astrocytes were identified using a GFAP antibody, and images were then analyzed. The colors on the images indicate different morphological features: White = cell body, Blue = first-order branch, Green = second-order branch, Yellow = third-order branch, and Red = fourth-order branch; Red square = branch point; and Red circle = intersections. (**B**) The total length of the astrocytic processes from the ACC regions. The total length of the astrocytic processes in different groups was significantly increased compared with the sham NP group. (**C**) The cell body areas of astrocytes in the ACC. The cell body areas of the astrocytes in different groups were increased significantly compared with the sham NP group. (**D**) The number of dendrites in different groups. There were significant increases in the number of primary (first-order) processes in experimental groups compared with the sham NP group. (**E**) The number of nodes in different groups. The numbers of nodes (second to fourth-order branch) in the experimental groups were significantly increased compared with the sham NP groups. Taken together, peripheral neuropathy induced astrocytic hypertrophy; however, MCS or ZIP did not interfere with this response. *P < 0.05 compared with sham NP.

We analyzed the morphology of the astrocytes to assess the total length and volume of processes combined with the cell body area as a measure of hypertrophy. The complexity of astrocytes was also assessed by counting the number of dendrites and node tips per astrocyte (Fig. 8A). The sum of astrocytic process lengths radiating from cell bodies to the end of processes was calculated for each astrocyte in the contralateral side of the ACC area (Fig. 8B, Sham NP, 332.22 ± 6.62; NP + Sham MCS, 493.33 ± 4.71; NP + MCS, 491.34 ± 12.01; NP + MCS + ZIP, 494.44 ± 7.28; ZP + MCS + Vehicle, 505.56 ± 8.98). To assess astrocytic hypertrophy, we measured the area of the cell body of the astrocytes in the ACC. The volume of the astrocytic processes also increased significantly in the contralateral side of the ACC cortex of nerve-injured animals compared with sham nerve-injury (Fig. 8C, Sham NP, 76.56 ± 3.85; NP + Sham MCS, 109.78 ± 2.59; NP + MCS, 115.33 ± 4.19; NP + MCS + ZIP, 115 ± 3.13; ZP + MCS + Vehicle, 106.67 ± 12.97). We also quantified the number of dendrites and nodes (Fig. 8D, Sham NP, 7.56 ± 0.29; NP + Sham MCS, 12.45 ± 0.82; NP + MCS, 11.22 ± 0.32; NP + MCS + ZIP, 10.78 ± 0.46; ZP + MCS + Vehicle, 11.88 ± 0.54 and Fig. 8E, Sham NP, 14.33 ± 0.65; NP + Sham MCS, 23.56 ± 0.63; NP + MCS, 23.11 ± 0.54; NP + MCS + ZIP, 21.78 ± 0.66; ZP + MCS + Vehicle, 22 ± 0.69). When compared with the sham group rats, we observed significant differences in all groups. Peripheral neuropathy induced astrocytic hypertrophy; however, neither MCS nor ZIP affected this response.

## Discussion

Our results show that nerve injury-induced neuropathic pain could contribute to the plastic neuronal changes in the ACC and leads to alterations in astrocytic processes and structural changes in the ACC. Although repetitive MCS in neuropathic pain reduces abnormal pain-like behavior, it was difficult to observe significant changes induced by MCS between neuropathic rats and other experimental rats. The phenomenon of synaptic plasticity accompanied by meta-plasticity affects the astrocytic regulation of synaptic transmission. This phenomenon depends on alterations in synaptic plasticity related to LTP in the ACC. These structural synaptic changes may account for the observed changes in synaptic regulation due to glial-neuronal interactions.

### Effects of repetitive MCS on neuropathic pain

Since its development in the early 1990s, MCS has been used to treat a variety of neuropathic pain syndromes^13, 26^. Initially used for thalamic pain, MCS has also been used to treat many types of pain typically resistant to common methods of pain management. Examples of other types of pain treated with MSC include phantom limb pain, postherpetic neuralgia, brachial plexus avulsion, post-stroke pain, Wallenberg syndrome, complex regional pain syndrome, pain secondary to multiple sclerosis, spinal cord injury pain, and post-traumatic brain injury pain^27^. The current evidence shows that a positive response is achieved in 44–100% of MCS-treated patients, and long-term benefits have been achieved in 0–100% of those patients who responded to initial trials of stimulation. There is also evidence to indicate that this response to stimulation decreases over time in some patients, and “intensive programming” is necessary to recapture the pain relief from the therapy^28, 29^.

The results of our controlled MCS in different group trials confirm that 10 daily sessions of primary motor cortex (M1) stimulation, using epidural MCS, could be very effective for decreasing the mechanical threshold and increasing pain-like behavior in neuropathic pain without serious adverse events. After a single MCS session, analgesic effects occur immediately and last for several hours^30^. Repeated daily MCS sessions appear to increase and prolong the pain-reliving effects. The sustained analgesic effects of MCS were observed 2 days after repeated MCS and lasted more than 2 weeks after the end of motor cortex stimulation. The data presented here clearly demonstrate that it is possible to maintain the analgesic effects of MCS on neuropathic pain over a long period of time. The reason for these results may be due to MCS-induced plastic changes in neural cells in the brain, as the effect of MCS on pain modulation is highly associated with neural plasticity in the brain.

Recent studies of chronic neuropathy have focused on peripheral neuropathy and long-term synaptic plasticity in the spinal cord, subcortical areas, and cortical areas^18, 31^. Cellular and molecular studies using rodent models have shown that the activation of glia can also have beneficial effects, including the release and maintenance of anti-inflammatory factors that protect against neurotoxicity and restore normal pain signaling^32^. Current studies suggest that glial-neuron interactions play critical roles in the development and maintenance of neuropathic pain^33–35^. Some fundamental questions about glial-neuronal interactions in pain mechanisms have been addressed, such as “What are the signals leading to central glial activation after injury?” and “How do glial cells affect central nervous system neuronal activity and promote hyperalgesia?”^36^ Several studies have demonstrated that astrocytes utilize ionotropic and metabolic systems to regulate neuron-to-neuron communication, such as sodium, potassium, chloride, and hydrogen ions, as well as mGluR, and NMDA and AMPA receptors^37, 38^. These findings imply that glial activation could be initiated by neurotransmitters and neuromodulators through glial-neuronal interactions and signaling^39^. Astrocytes are a key trigger for primary somatosensory circuit rewiring and contribute to the mechanical allodynia in neuropathic rats^7^. Nevertheless, many studies have been limited to the spinal level and the role of astrocytes in the brain is not well understood.

In our study, MCS-induced analgesia was completely blocked by ZIP injection in the ACC of MCS-treated neuropathic rats. The concomitant glial-neuronal activation and LTP in the ACC during the MCS period could be important in the context of hypersensitivity of the pain response. Here, we found a relationship between mechanical allodynia and glial-neuronal cellular activation in the ACC. Our findings strongly suggest that the reduced neuropathic pain produced by repetitive MCS may due to the attenuation of nerve injury-induced activation of glial-neuronal interactions. However, this study did not clearly demonstrate MCS-induced glial-neuronal activation. Nevertheless, the completely suppressed MCS effect by ZIP injection suggests that ZIP could affect the newly generated glial-neuronal synaptic changes after repetitive MCS. ZIP is considered to be a candidate inhibitor of the atypical PKMζ, which is thought to be responsible for maintaining the late phase of LTP^40^. Previous studies using ZIP have shown that ZIP effectively inhibited the production of LTP in several brain regions, such as the hippocampus, amygdala, insular cortex and sensorimotor cortex^31, 41^. When LTP is generated, the number of surrounding astrocytes is increased to supply enough energy for the newly generated synapses^6^. Therefore, the degree of astrocytic hypertrophy, which can be determined by measuring the number of and assessing shape of astrocytes, can be used as direct or indirect evidence of activated synaptic plasticity^38^. In this study, our hypothesis is that the MCS–induced incertothalamic pathway activation produces pain inhibition related to LTP in the ACC. As our results showed, the MCS-induced analgesic effects were blocked by ZIP treatment. This result suggests that the ZIP may inhibit the maintenance of MCS-induced LTP in the ACC. Furthermore, this reduction of MCS-induced LTP could influence nociceptive neurotransmission after MCS.

### Alterations in glial-neuronal interactions after neuropathic pain

Injury to the somatosensory nervous system can produce chronic neuropathic pain characterized by abnormal sensations, such as allodynia and hyperalgesia. Recent studies have reported that glial activation is an integral part of pain pathogenesis, and the activation of astrocytes is directly involved in pain hypersensitivity^7, 42^. After nerve damage, the pathogenesis of chronic pain involves a series of interactions between cells, such as neurons, astrocytes, and microglia, in the CNS. In an animal model of neuropathic pain, synaptic plasticity in the ACC was potentiated 1–2 weeks after nerve injury at the time of the development of allodynia^41^. Activity-dependent gene expression produces late LTP in the hippocampus. Furthermore, increased neuronal excitability and synaptic potentiation in the ACC, as evidenced in a model of chronic pain, produce a long-lasting enhancement of synaptic responses in the ACC to peripheral stimulation^40^. Consistent with this result, we suggest that neuropathic pain is linked to the expression of LTP in the ACC. Furthermore, glial cells are no longer considered passive bystanders in neuronal brain circuits^5, 43^. Not only are they required for housekeeping and brain metabolism, they are active participants in regulating the physiological function and plasticity of brain circuits. Regulation of ionic gradients by glial cells can underlie the bistability of neurons and can modulate the fidelity of synaptic transmission. Grafting of human glial progenitor cells in mouse forebrain enhanced plasticity and improved the behavioral performance, suggesting that astrocytes have evolved to cope with information processing in more complex brains^44^. Taken together, current evidence strongly suggests that glial cells are essential contributors to information processing in the brain^44^. However, additional research is required to understand the functions of the current strategy of inhibiting all glial-neuronal interactions without fully understanding its physiological or pathological roles.

### MCS-induced alterations in astrocytes in the ACC

We studied the potential factors influencing the analgesic effect of repetitive MCS. In a previous study, regulated activation of the zona incerta-posterior thalamus incertothalamic pathway by MCS could reduce the mechanical threshold after nerve injury^30^. Although a lot of evidence from animal and human studies has supported the pain modulation effects of MCS, additional studies are required to validate the evidence of potential factors on MCS-related pain modulation. Several hypotheses have been proposed to explain how MCS leads to pain reduction^10, 13, 17^. We hypothesized that MCS modulates corticothalamic connections, and these connections in turn produce synaptic plasticity in the ACC. Previous studies investigating brain activation using fMRI showed that MCS attenuated signals in the prefrontal cortex (PFC), ACC, and primary somatosensory cortex (S1)^31, 45^. Pagano *et al*.^46^ showed an increase in ACC activation in neuropathic rats after MCS-induced analgesia, corroborating the idea that the neuroplasticity in the ACC is crucial to the MCS-induced analgesic effect. Furthermore, several cortical nociceptive processing correlated structures, such as the S1, secondary somatosensory cortex (S2), anterior insular, cingulate, and dorsolateral prefrontal cortices are activated by MCS^45^. This result suggests that MCS-induced plastic changes could affect the suppression of pain related to the brain matrix. Although MCS could affect signaling in neural networks in the brain and modulate the excitability of neuronal activity, the mechanism for the pain relief induced by MCS remains hypothetical. The current hypothesis for the underlying mechanism of MCS is that MCS modifies the excitability of neuronal activity involved in the neural circuits responsible for pain processing and perception^12, 47^. Even though we have shown detailed evidence of increased dendrites in astrocytes, morphological alterations of glial-neuronal cells, and expression of neurotransmitters and proliferation of astrocytes in the MCS group after nerve injury, we must consider that the present findings do not provide distinct evidence linking the neuropathic pain-induced cortical neuronal cell changes and repetitive MCS-induced alterations of astrocytic processes and structural changes.

We report MCS-induced pain modulation in neuropathic pain rats and glial-neuronal interactions and morphological changes after nerve injury in animals, and these results provide sufficient evidence to support the glial-neuronal interaction mechanism for pain pathogenesis. Understanding how MCS affects multiple cortical regions downstream of each specific target should enable important fundamental advances in the use of this therapeutic modality. Greater knowledge of the network effects of MCS will permit the tailoring of treatment based on biomarkers related to these effects to increase therapeutic efficacy.

## Methods

### Animals

Male Sprague–Dawley rats (Koatech, Pyeongtaek, Korea; 200–220 g) were housed in ventilated plastic cages with soft bedding and maintained on a 12/12 hour light/dark cycle (lights on at 07:00) at a constant temperature (22 ± 2 °C) and humidity (50 ± 10%) in an animal facility accredited by AAALAC International. The rats were fed standard rat chow and had access to tap water *ad libitum*. All animal experiments were performed in accordance with the guidelines for the ethical use of conscious animals in pain research published by the International Association for the Study of Pain and were approved by the Institutional Animal Care and Use Committee of Yonsei University Health System (protocol number 2016–0061). All efforts were made to minimize animal suffering and to reduce the number of animals used.

### Experimental design

The animals were evaluated in the nociceptive test for 2 weeks after the peripheral neuropathic injury or sham operation, which was performed in the right hind limb of the anesthetized rats. After 2 weeks, transdural electrodes and osmotic pumps were implanted over the motor cortex contralateral to nerve injury. From one day after implantation, the nociceptive tests were performed before (pre) and after (post) MCS in awake animals for 10 days. After the last behavioral test, the animals were anesthetized and tissue removed for western blot and immunohistochemistry assays.

### Animal model of neuropathic pain

The rats were anesthetized with sodium pentobarbital (50 mg/kg, i.p.). The respiratory rate, corneal reflex, and tail pinch response were monitored to ensure that animals were sufficiently anesthetized. The surgical procedure was performed following the methods described in our previous report^48^. A segment of the sciatic nerve was exposed, and three major divisions of the sciatic nerve (common peroneal, tibial, and sural nerves) were clearly separated. Then, the tibial and sural nerves were tightly ligated and then transected; meanwhile, the common peroneal nerve was left intact. Complete hemostasis was confirmed, and the wound was closed with muscle and skin sutures. The sham control group underwent the same operation but without any nerve damage.

### MCS

The deeply anesthetized animals were attached to a stereotaxic frame and placed on a thermo-regulated heating pad. A local anesthetic (2% lidocaine) was applied to the incision sites before surgery began. The bone overlying the M1 was removed, and custom-made insulated bipolar platinum electrodes (height, 70 μm; exposed tip, 50 μm; distance between electrodes, 500 μm) were applied epidurally above the M1 at stereotaxic coordinates determined from previous MCS experiments (anterior, 1.8 mm; lateral, 2 mm), contralateral to the nerve injury site^49^. The MCS electrodes were held in place using 2 bone screws and dental cement. The electrodes were connected to a stimulator (A385, WPI, Sarasota, FL, USA) for the behavioral test during repetitive MCS. In all experiments, the M1 was stimulated continuously at 50 μA and 50 Hz for 300 ms pulses^30, 49^. The sham MCS group underwent the same procedure without any electrical stimulation.

### Long-term administration of ZIP into the ACC

To quantify the plastic changes in the ACC after peripheral nerve injury and MCS-induced neural plasticity in the ACC, an osmotic minipump (Model 1007D, infusion flow rate: 0.5 μl/hour in Infusion Kit 1; Alzet Osmotic Pumps, Cupertino, CA, USA) was stereotaxically implanted into the contralateral ACC of the nerve injury (coordinates for the anterior cingulate cortex: AP = +1.7, ML = +0.8, DV = 1.5 from bregma) according to the atlas of Paxinos and Watson^50^. A cannula was connected to the osmotic minipump through PE-20 tubing filled with a 10 nmol/μL solution of ζ-pseudosubstrate inhibitory peptide (ZIP), a selective inhibitor of PKMζ, or saline. The implanted pump delivered solutions into the brain continuously for 7 days. The rats received either ZIP or vehicle injection via the implanted osmotic pump during the MSC session.

### Mechanical allodynia testing

To assess mechanical withdrawal thresholds and latency, an electrical von Frey (38450, Ugo Basile, Comerio, Italy) was used. To minimize anxiety, the animals were acclimated to the behavioral apparatus 10 minutes before testing. The filament was applied 7 times to the receptive surface of the hind paws. The positive responses to mechanical stimulation included licking, sudden withdrawal, and biting of the ipsilateral paw. The withdrawal latencies were defined as the time between the peripheral stimulation with a von Frey filament and the responses to the stimulation. The withdrawal thresholds and latencies were assessed before neuropathic surgery, at days 1, 4, 7, and 14 post operation, and at daily intervals pre- and post-MCS.

### Immunohistochemistry (IHC)

For IHC, the rats were perfused with 200 ml 0.9% NaCl and 4% paraformaldehyde in sodium phosphate buffer, and the brains were excised and incubated with 4% paraformaldehyde for 24 hours before freezing in an embedding compound on dry ice. The frozen brains were cut with a cryostat (HM525, Thermo Scientific, Waltham, MA, USA) at a thickness of 30 μm. The sections were incubated overnight at 4 °C with the following primary antibodies: rabbit anti-GFAP (1:500; catalog ab7260; Abcam); and mouse anti-NeuN (1:100; catalog MAB377; Millipore) and then washed with PBS and further incubated for 2 hours at room temperature with Alexa Fluor 488– and Cy^tm^ 3-conjugated AffiniPure F(ab’)_2_ Fragment Donkey Anti-Mouse IgG secondary antibodies (all from Jackson ImmunoResearch, 1:200). DAPI was used for counterstaining. The immunofluorescence images were obtained using a confocal microscope with AxioScan Z1 (Zeiss, Goettingen, Germany) and LSM700 (Zeiss, Goettingen, Germany). The number of cells with colocalized expression of NeuN and GFAP were quantified. Briefly, 12 μm-thick confocal Z-stacks of the synaptic zone in the ACC and M1 cortex were imaged. The maximum projections of 3 consecutive optical sections were generated from the original Z-stack. Three image stacks per rat (n = 4 rats/group) were used for analyses.

### Western blotting

To collect ACC samples, the animals were anesthetized with enflurane and decapitated. The ipsilateral and contralateral ACCs were quickly isolated and transferred to a deep freezer. The extracted samples were stored at −70 °C. For protein extraction, the samples were homogenized by sonication in lysis buffer (Proprep, iNtRON Biotechnology Inc., Seongnam, Korea) containing phosphatase inhibitor (PhosStop, Roche, Penzberg, Germany). The samples were centrifuged at 22,250 × g for 10 minutes at 4 °C, supernatants were collected, and the total protein concentration of lysates was assessed using a spectrophotometer (ND-1000, NanoDrop Technologies Inc., Wilmington, DE, USA). The brain tissue extracts containing 10 μL of protein per well were denatured and separated on 10% Bis-Tris gels (Bio-Rad, Hercules, CA, USA) for the detection of GFAP and NeuN. The proteins were transferred onto polyvinylidene difluoride (PVDF) membranes (GE Healthcare, Buckinghamshire, UK). The membranes were blocked in 5% skim milk in TBS with Tween-20 for 1 hour and incubated in primary antibodies overnight on a rocking platform at 4 °C. The primary antibodies against GFAP (1:5,000, Cell Signaling Technology, Beverly, MA, USA), NeuN (1:2,000, Cell Signaling Technology), and β-actin (1:2,000, Cell Signaling Technology), which was used as a loading control, were used for western blot analyses. On the following day, the membranes were incubated with the appropriate secondary antibodies for 2 hours, and horseradish peroxidase activity was visualized using a chemiluminescent substrate (ECL Prime western blotting detection reagent, GE Healthcare) and processed with a local allocation system (LAS) (ImageQuant LAS 4000 Mini, GE Healthcare). The intensities of the GFAP and NeuN bands were normalized to the intensity of the β-actin band.

### Quantification of astrocyte morphology

All samples were coded and analyzed randomly by a researcher blinded to the animal number and condition. Images of non-overlapping fields in the ACC region sections were captured by fluorescence microscopy using a confocal microscope with AxioScan Z1 (Zeiss, Goettingen, Germany) and LSM700 (Zeiss, Goettingen, Germany). The measurement of astrocytic hypertrophy was described in detail in a previous study^51^. An average of 10 astrocytes in the ACC region from each animal with well-defined cell bodies and processes were chosen for analysis. The chosen cells were fully intact and did not have processes that touched the edges of the field. The resulting files generated by 2D tracing were analyzed using the Neurolucida Explorer program (MBF Bioscience, Williston, VT, USA), generating data of morphological measurements including arbor length, cell body area, number of dendrites, and number of nodes (branching points). A schematic of the astrocytes and parameters is shown in Fig. 8A.

### Statistics

Data are presented as the mean ± standard error of the mean (SEM). Statistical tests were performed using a 2-tailed, unpaired t-test (2 variables) or one-way ANOVA followed by Dunnett’s post hoc multiple comparison test to determine significance. To confirm the significant correlation between the daily pre- and post-MCS, Spearman correlation coefficient tests were performed at the daily session of MCS (where 1 is total positive linear correlation, 0 is no linear correlation, and −1 is total negative linear correlation). In all cases, a *P* value less than 0.05 was considered statistically significant. We excluded animals from the analysis when they showed any abnormality (e.g., poor general condition, loss of resin, or brain damage during the craniotomy) prior to or during the experiments.

## Electronic supplementary material

Supplymentary Fig 1

## References

[1] Mika J (2009). Differential activation of spinal microglial and astroglial cells in a mouse model of peripheral neuropathic pain. Eur J Pharmacol.

[2] Mika J, Zychowska M, Popiolek-Barczyk K, Rojewska E, Przewlocka B (2013). Importance of glial activation in neuropathic pain. Eur J Pharmacol.

[3] Tsuda M, Masuda T, Tozaki-Saitoh H, Inoue K (2013). Microglial regulation of neuropathic pain. J Pharmacol Sci.

[4] Yi M, Zhang H (2011). Nociceptive memory in the brain: cortical mechanisms of chronic pain. J Neurosci.

[5] Bernardinelli Y, Muller D, Nikonenko I (2014). Astrocyte-synapse structural plasticity. Neural Plast.

[6] Barker AJ, Ullian EM (2010). Astrocytes and synaptic plasticity. Neuroscientist.

[7] Kim SK (2016). Cortical astrocytes rewire somatosensory cortical circuits for peripheral neuropathic pain. The Journal of clinical investigation.

[8] Gao YJ, Ji RR (2010). Targeting astrocyte signaling for chronic pain. Neurotherapeutics.

[9] Tsubokawa T, Katayama Y, Yamamoto T, Hirayama T, Koyama S (1991). Chronic motor cortex stimulation for the treatment of central pain. Acta Neurochir Suppl (Wien).

[10] Kim, J. *et al*. Motor cortex stimulation and neuropathic pain: how does motor cortex stimulation affect pain-signaling pathways? *J Neurosurg*, 1–11, doi:10.3171/2015.1.jns14891 (2015). 10.3171/2015.1.JNS14891 26274988

[11] Garcia-Larrea L, M. J. & Peyron R. Thalamocingulate mechanisms of precentral cortex stimulation for central pain. In: Vogt B, editor. Cingulate Neurobiology and Disease. 437–465 (2009).

[12] DosSantos MF, Ferreira N, Toback RL, Carvalho AC, DaSilva AF (2016). Potential Mechanisms Supporting the Value of Motor Cortex Stimulation to Treat Chronic Pain Syndromes. Frontiers in neuroscience.

[13] Sokal P (2015). Motor cortex stimulation in patients with chronic central pain. Adv Clin Exp Med.

[14] Khedr EM (2005). Longlasting antalgic effects of daily sessions of repetitive transcranial magnetic stimulation in central and peripheral neuropathic pain. J Neurol Neurosurg Psychiatry.

[15] Antal A, Terney D, Kuhnl S, Paulus W (2010). Anodal transcranial direct current stimulation of the motor cortex ameliorates chronic pain and reduces short intracortical inhibition. J Pain Symptom Manage.

[16] Siebner HR (2004). Preconditioning of low-frequency repetitive transcranial magnetic stimulation with transcranial direct current stimulation: evidence for homeostatic plasticity in the human motor cortex. J Neurosci.

[17] Hawken, E. R. *et al*. Transcranial Magnetic Stimulation of the Supplementary Motor Area in the Treatment of Obsessive-Compulsive Disorder: A Multi-Site Study. *Int J Mol Sci* **17**, doi:10.3390/ijms17030420 (2016). 10.3390/ijms17030420 PMC4813271 27011177

[18] Zhuo M (2008). Cortical excitation and chronic pain. Trends Neurosci.

[19] Yamashita A (2014). Astrocytic activation in the anterior cingulate cortex is critical for sleep disorder under neuropathic pain. Synapse.

[20] Lu Y, Zhu L, Gao YJ (2011). Pain-related aversion induces astrocytic reaction and proinflammatory cytokine expression in the anterior cingulate cortex in rats. Brain Res Bull.

[21] Chen FL (2012). Activation of astrocytes in the anterior cingulate cortex contributes to the affective component of pain in an inflammatory pain model. Brain Res Bull.

[22] Fields RD, Stevens-Graham B (2002). New insights into neuron-glia communication. Science.

[23] Giaume C, Tabernero A, Medina JM (1997). Metabolic trafficking through astrocytic gap junctions. Glia.

[24] Laming PR (2000). Neuronal-glial interactions and behavior. Neurosci Biobehav Rev.

[25] Cha MH, Nam TS, Kwak Y, Lee H, Lee BH (2012). Changes in cytokine expression after electroacupuncture in neuropathic rats. Evid Based Complement Alternat Med.

[26] Lefaucheur JP (2009). Motor cortex stimulation for the treatment of refractory peripheral neuropathic pain. Brain.

[27] Monsalve GA (2012). Motor cortex stimulation for facial chronic neuropathic pain: A review of the literature. Surg Neurol Int.

[28] Henderson JM, Boongird A, Rosenow JM, LaPresto E, Rezai AR (2004). Recovery of pain control by intensive reprogramming after loss of benefit from motor cortex stimulation for neuropathic pain. Stereotact Funct Neurosurg.

[29] Smith H (2001). Motor cortex stimulation for neuropathic pain. Neurosurg Focus.

[30] Lucas JM, Ji Y, Masri R (2011). Motor cortex stimulation reduces hyperalgesia in an animal model of central pain. Pain.

[31] Zhuo M (2014). Long-term potentiation in the anterior cingulate cortex and chronic pain. Philos Trans R Soc Lond B Biol Sci.

[32] Milligan ED, Watkins LR (2009). Pathological and protective roles of glia in chronic pain. Nat Rev Neurosci.

[33] Scholz J, Woolf CJ (2007). The neuropathic pain triad: neurons, immune cells and glia. Nat Neurosci.

[34] Ji RR, Berta T, Nedergaard M (2013). Glia and pain: is chronic pain a gliopathy?. Pain.

[35] Bradesi S (2010). Role of spinal cord glia in the central processing of peripheral pain perception. Neurogastroenterol Motil.

[36] Ren K, Dubner R (2008). Neuron-glia crosstalk gets serious: role in pain hypersensitivity. Curr Opin Anaesthesiol.

[37] Simard M, Nedergaard M (2004). The neurobiology of glia in the context of water and ion homeostasis. Neuroscience.

[38] Ota Y, Zanetti AT, Hallock RM (2013). The role of astrocytes in the regulation of synaptic plasticity and memory formation. Neural Plast.

[39] Tiwari V, Guan Y, Raja SN (2014). Modulating the delicate glial-neuronal interactions in neuropathic pain: promises and potential caveats. Neurosci Biobehav Rev.

[40] Wei F, Zhuo M (2001). Potentiation of sensory responses in the anterior cingulate cortex following digit amputation in the anaesthetised rat. J Physiol.

[41] Li XY (2010). Alleviating neuropathic pain hypersensitivity by inhibiting PKMzeta in the anterior cingulate cortex. Science.

[42] Nam Y (2016). Reversible Induction of Pain Hypersensitivity following Optogenetic Stimulation of Spinal Astrocytes. Cell reports.

[43] Perea G, Sur M, Araque A (2014). Neuron-glia networks: integral gear of brain function. Frontiers in cellular neuroscience.

[44] Hoogland, T. & Parpura, V. Editorial: The role of glia in plasticity and behavior. *Frontiers in cellular neuroscience* **9**, doi:10.3389/fncel.2015.00356 (2015). 10.3389/fncel.2015.00356 PMC4563082 26441527

[45] Jiang L (2014). Motor cortex stimulation suppresses cortical responses to noxious hindpaw stimulation after spinal cord lesion in rats. Brain Stimul.

[46] Pagano RL (2011). Transdural motor cortex stimulation reverses neuropathic pain in rats: a profile of neuronal activation. European journal of pain (London, England).

[47] Zaghi S, Heine N, Fregni F (2009). Brain stimulation for the treatment of pain: A review of costs, clinical effects, and mechanisms of treatment for three different central neuromodulatory approaches. Journal of pain management.

[48] Lee BH, Won R, Baik EJ, Lee SH, Moon CH (2000). An animal model of neuropathic pain employing injury to the sciatic nerve branches. NeuroReport.

[49] Cha M, Ji Y, Masri R (2013). Motor cortex stimulation activates the incertothalamic pathway in an animal model of spinal cord injury. J Pain.

[50] G. Paxinos, C. W. *The Rat Brain in Stereotaxic Coordinates* (Academic Press, 1986).

[51] Lee, K. *et al*. Aerosol-induced brucellosis increases TLR-2 expression and increased complexity in the microanatomy of astroglia in rhesus macaques. *Frontiers in Cellular and Infection Microbiology* **3**, doi:10.3389/fcimb.2013.00086 (2013). 10.3389/fcimb.2013.00086 PMC3844859 24350061

