# Supplementary material for: Repetitive motor cortex stimulation reinforces the pain modulation circuits of peripheral neuropathic pain
Source: Sci Rep. 2017 Aug 11;7:7986. doi: 10.1038/s41598-017-08208-2 (PMC5554204; doi:10.1038/s41598-017-08208-2)
Supplement: Supplementary file 1 — Supplymentary Fig 1 [file 41598_2017_8208_MOESM1_ESM.pdf]

Repetitive motor cortex stimulation reinforces the pain modulation circuits of peripheral neuropathic pain

Myeounghoon Cha[1], Sun Woo Um[1,2], Minjee Kwon[1,2], Taick Sang Nam[1], Bae Hwan Lee[1,2*]

[1] Department of Physiology, Yonsei University College of Medicine, Seoul 03722, Republic of Korea

[2] Brain Korea 21 PLUS Project for Medical Science, Yonsei University College of Medicine, Seoul 03722, Republic of Korea

* Corresponding Author

Bae Hwan Lee, PhD

Department of Physiology, Yonsei University College of Medicine

50-1, Yonsei-ro, Seodaemun-gu

Seoul 03722, Korea

Tel.: +82.2.2228.1711

Fax: +82.2.393.0203

E-mail address: bhlee@yuhs.ac

Keywords: motor cortex stimulation, pain modulation, astrocyte, anterior cingulate cortex

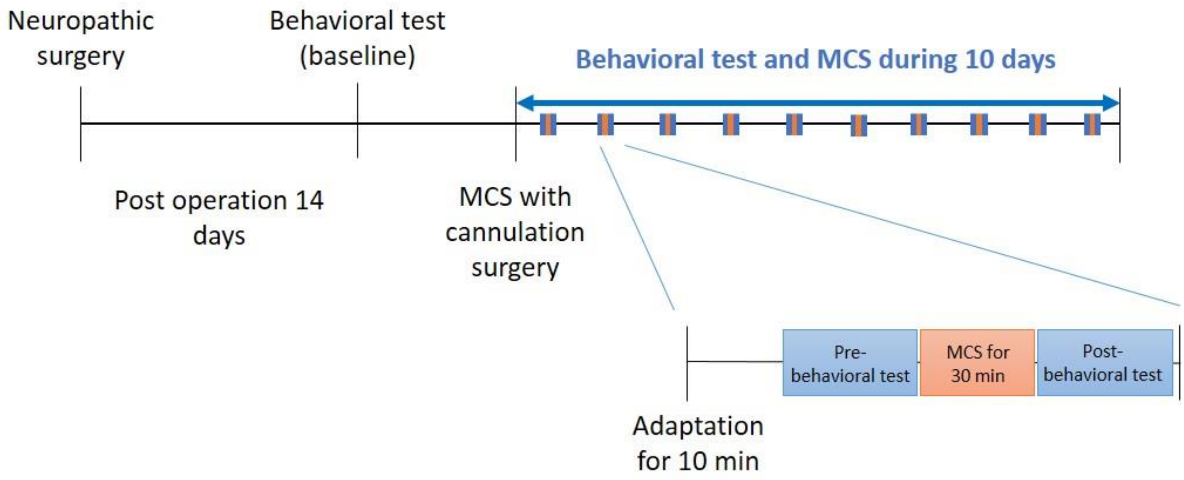

Supplementary Figure 1

Schematic diagram of behavioral tests and MCS. Pain behavior was tested 2 weeks after the peripheral neuropathy or sham operation. The transdural electrodes and osmotic pumps were implanted over the motor cortex contralateral to nerve injury. From one day after implantation, the nociceptive changes were measured before (pre) and after (post) MCS in awake animals for 10 days. After the last behavioral test, the animals were anesthetized and tissue removed for western blot and immunohistochemistry assays.
